# Supplementary material for: Using drivers and transmission pathways to identify SARS-like coronavirus spillover risk hotspots
Source: Nat Commun. 2023 Oct 27;14:6854. doi: 10.1038/s41467-023-42627-2 (PMC10611769; doi:10.1038/s41467-023-42627-2)
Supplement: Supplementary file 1 — Supplementary Information [file 41467_2023_42627_MOESM1_ESM.pdf]

# Supplementary Information

## Using drivers and transmission pathways to identify SARS-like coronavirus spillover risk hotspots

*Renata L. Muylaert[1*], David A Wilkinson[2], Tigga Kingston[3], Paolo D'Odorico[4], Maria Cristina Rulli[5], Nikolas Galli[5], Reju Sam John[6], Phillip Alviola[7], David T. S. Hayman[1]*

[1] *Massey University, Palmerston North, New Zealand*

[2] *UMR ASTRE, CIRAD, INRAE, Université de Montpellier, Plateforme Technologique CYROI, Sainte-Clotilde, La Réunion France*

[3] *Department of Biological Sciences, Texas Tech University, Lubbock, TX, U.S.A.*

[4] *Department of Environmental Science, Policy, and Management, University of California, Berkeley, Berkeley, CA, U.S.A.*

[5] *Department of Civil and Environmental Engineering, Politecnico di Milano, Milan, Italy*

[6] *Department of Physics, Faculty of Science, University of Auckland, Auckland, New Zealand*

[7] *Institute of Biological Sciences, University of the Philippines- Los Banos, Laguna, Philippines*

*Corresponding author: **R.deLaraMuylaert[at]massey.ac.nz**

**This file includes:**

Supplementary Table 1

Supplementary Table 2

Supplementary Table 3

Supplementary Figures 1-11

**Supplementary Table 1:** Average time in hours to reach healthcare in areas where high emergent risk co-occurred with areas far from healthcare (third upper quantile). In Scenario 3, the average time to reach healthcare is considerably higher.

| Scenario | High-risk areas (N) far from healthcare | Average time to reach healthcare (hours, minutes, seconds) | min | max | SD |
|---|---|---|---|---|---|
| Scenario 1 | 26 | 3.93 (3 h 55 min 48 s) | 3.13 | 4.13 | 0.62 |
| Scenario 2 | 78 | 4.06 (4 h 03 min 36 s) | 3.13 | 4.43 | 0.81 |
| Scenario 3 | 236 | 4.81 (4 h 48 min 36 s) | 3.13 | 5.22 | 1.81 |
| Scenario 4 | 59 | 4.05 (4 h 03 min 00 s) | 3.13 | 4.33 | 0.82 |

**Supplementary Table 2: Hypothesized risk indicators informing the transmission scenarios, their rationale for inclusion, description, and sources.** Original rasters were warped to 0.25 decimal degrees and World Geodetic System (WGS 84).

| **Higher-level indicator** | **Univariate spatial layers** | **Rationale for inclusion** | **References** | **Spatial layer details** | **Spatial layer source** |
|---|---|---|---|---|---|
| Landscape change (all scenarios). | Three layers were used, representing anthropogenic stressor intensities of: Built up area; Energy and mining; Agriculture and harvest. | Coronavirus shedding may be higher in human-dominated areas. Mining and agricultural areas are a signal of human activity even when population counts are low and can represent the margins where natural host habitat may be closer to human encounters. | [1] | Summarizes land use intensity by human modification in 2017 (~1 km). | [2] |
| Landscape change (all scenarios). | Forest quality. | Emerging infectious disease risk is elevated in forested tropical regions experiencing land-use changes and where wildlife | [3] | Forest landscape integrity index, where highest values indicate highest quality (low=0, high=10) for 2019 (~1 km). It | [4] |

| | | | | | |
|---|---|---|---|---|---|
| | | biodiversity (mammal species richness) is high. | | is based on inferred and observed human pressures (infrastructure, agriculture, tree cover loss) and loss of forest connectivity. | |
| Landscape change (all scenarios). | Risk of cover loss based on threats and dynamics. | Theory on land-use induced spillover; Agricultural land-uses exacerbate many infectious diseases in Southeast Asia (malaria, Schistosomiasis, Spotted fever, hookworms). | [5–7] | It informs the risk of a forest becoming removed in the future (transition potential, ~1 km), based on neural network models using historical data (2001-2014) from low (0) to high risk (1). Here we use continental model outcomes and not global, as the regional model estimates for Asia had better performance than the global model. | [8] https://futureclimates.conservation.org/riskstreecoverloss.html |

| | | | | | |
|---|---|---|---|---|---|
| Potential secondary host (Scenario 2, Scenario 4). | Pigs. | Coronaviruses with origins tracing to bats causing disease in pigs. Sporadic infections cannot be excluded, but large-scale SARS-CoV-2 transmission among pigs is unlikely [9]. Respiratory illness symptoms have been associated with human contact with wildlife and livestock [10]. | [9,11] | Areal-weighted GLW model ('Aw.tif' files) from GLW3 Gilbert's livestock of the world estimates for 2010 (~10 km). This layer's original data spreads individuals of a census polygon evenly, so the density of animals in each pixel corresponds to the average number of animals/km$^2$ of suitable land in the census unit. | [12] |
| Potential secondary host (Scenario 2, Scenario 4). | Cattle, bovid livestock. | Recent evidence from Germany. Concerns the potential for anthropozoonotic infections of cattle reported as the presence of a | [13,14] | Areal-weighted GLW model ('Aw.tif' files) from Gilbert's livestock of the world estimates for 2010 (~10 km). This layer's original | [12] |

| | | | | | |
|---|---|---|---|---|---|
| | | preexisting coronavirus did not protect from infection with another betacoronavirus in a study. Also, multiple infections of individual animals might lead to recombination events between a SARS-like coronavirus and Bovine Coronavirus, a phenomenon already described for other pandemic coronaviruses. | | data spreads individuals of a census polygon evenly, so the density of animals in each pixel corresponds to the average number of animals/km$^2$ of suitable land in the census unit. Results with all bovid livestock in the supplements (buffalo, cattle, goat, sheep). | |
| Potential secondary host (Scenario 3, Scenario 4). | Wild mammals minus known bat hosts. | Emerging infectious disease risk is elevated in forested tropical regions experiencing land-use changes and where wildlife biodiversity | [3,15] | IUCN data (~30 km), Search on 2022-04-04. Original Mollweide projection was warped to WGS84 in QGIS 3.24 after subtracting known | https://www.iucnredlist.org/resources/other-spatial-downloads#SR_2021_3 |

| | | | | | |
|---|---|---|---|---|---|
| | | (mammal species richness) is high. SARS-Cov-2 has been detected in wildlife (spillback events). | | bat host ranges. | |
| Primary host (all scenarios). | Average estimated number of species of known bat hosts. | Peak of sarbecovirus hosts in Asia; Both the evolutionary and ecological aspects of emergence risk are higher in southeast Asia—a fact that will only become more relevant, as bats track shifting climates and exchange viruses with other species, creating a hotspot of elevated cross-species transmission unique to the region. Experimental evidence for bat | [16–19] | Average values used from the two sources. Sánchez et al. (2022) data (~1 km areas of habitat) was resampled to match Muylaert et al. (2022) resolution (0.25 dd). | [16,17] |

| | | | | | |
|---|---|---|---|---|---|
| | | (SARS-like) coronaviruses viruses infecting human cells | | | |
| Exposure (all scenarios). | Human population counts. | Population size is a crucial factor for SARS-like disease spread. | [20,21] | Worldpop unconstrained global mosaics of population counts for 2020 (1 km spatial resolution). | https://hub.worldpop.org/geodata/listing?id=64 |
| Detection and spread in humans (not in the scenarios). | Travel time to healthcare. | City remoteness and hence access to healthcare are key to understanding zoonotic disease outbreaks. They can be used to understand early detection and connectivity. | [22] | Travel time to healthcare (motorized minutes, 1 km spatial resolution). This layer provides travel times to a nearest geolocated hospital or clinic. Hospital and clinic definitions vary among countries, but they assume they are: Fixed facilities providing urgent or emergency | [23] |

| | | | | | |
|---|---|---|---|---|---|
| | | | | medical care with an entry subtype indicating they were a hospital or clinic, that were open in August 2019. Mobile or temporary clinics for providing healthcare in remote areas are not considered. Weiss et al. (2020) report that travel time estimates were generally accurate. All data is recent, including datasets that were published in 2019. The authors emphasize that Google Maps and OSM data are frequently updated with robust quality controls. In terms of sources of data and coverage, the | |

| | | | | | |
|---|---|---|---|---|---|
| | | | | Google dataset provided the best source of information on facility location in Asia.<br>Data coverage for healthcare varies by country. We assume that there is good data completeness in Asia, and Google had the best healthcare facility data sources for Asian countries. China has the largest number of pixels with healthcare facilities in the world (Supplementary Table 3), followed by other Asian countries considered in our analysis, such as India, Indonesia, Thailand, and | |

| | | | | | |
|---|---|---|---|---|---|
| | | | | Malaysia. | |

**Supplementary Table 3:** Travel time and healthcare facility pixel count for the region of study based on the source data set[23]. A pixel is counted when it contains one healthcare facility or more.

| Country | People per hospitals and clinics pixel | Hospitals and clinics pixel count | % Hospitals and clinics pixel count per world total |
|---|---:|---:|---:|
| **World total** | **19200** | **379231** | **100.000%** |
| China | 25900 | 53451 | 14.095% |
| India | 52200 | 24136 | 6.364% |
| Indonesia | 14800 | 17014 | 4.486% |
| Thailand | 6900 | 9735 | 2.567% |
| Malaysia | 10800 | 2769 | 0.730% |
| Philippines | 43200 | 2358 | 0.622% |
| Vietnam | 40100 | 2282 | 0.602% |
| Bangladesh | 131400 | 1208 | 0.319% |
| Sri Lanka | 25600 | 838 | 0.221% |
| Nepal | 54300 | 582 | 0.153% |
| Myanmar | 147500 | 339 | 0.089% |
| Singapore | 23000 | 252 | 0.066% |
| Cambodia | 80500 | 192 | 0.051% |
| Timor-East | 9300 | 128 | 0.034% |
| Lao PDR | 89400 | 76 | 0.020% |
| Bhutan | 33100 | 24 | 0.006% |
| Brunei | 36300 | 11 | 0.003% |

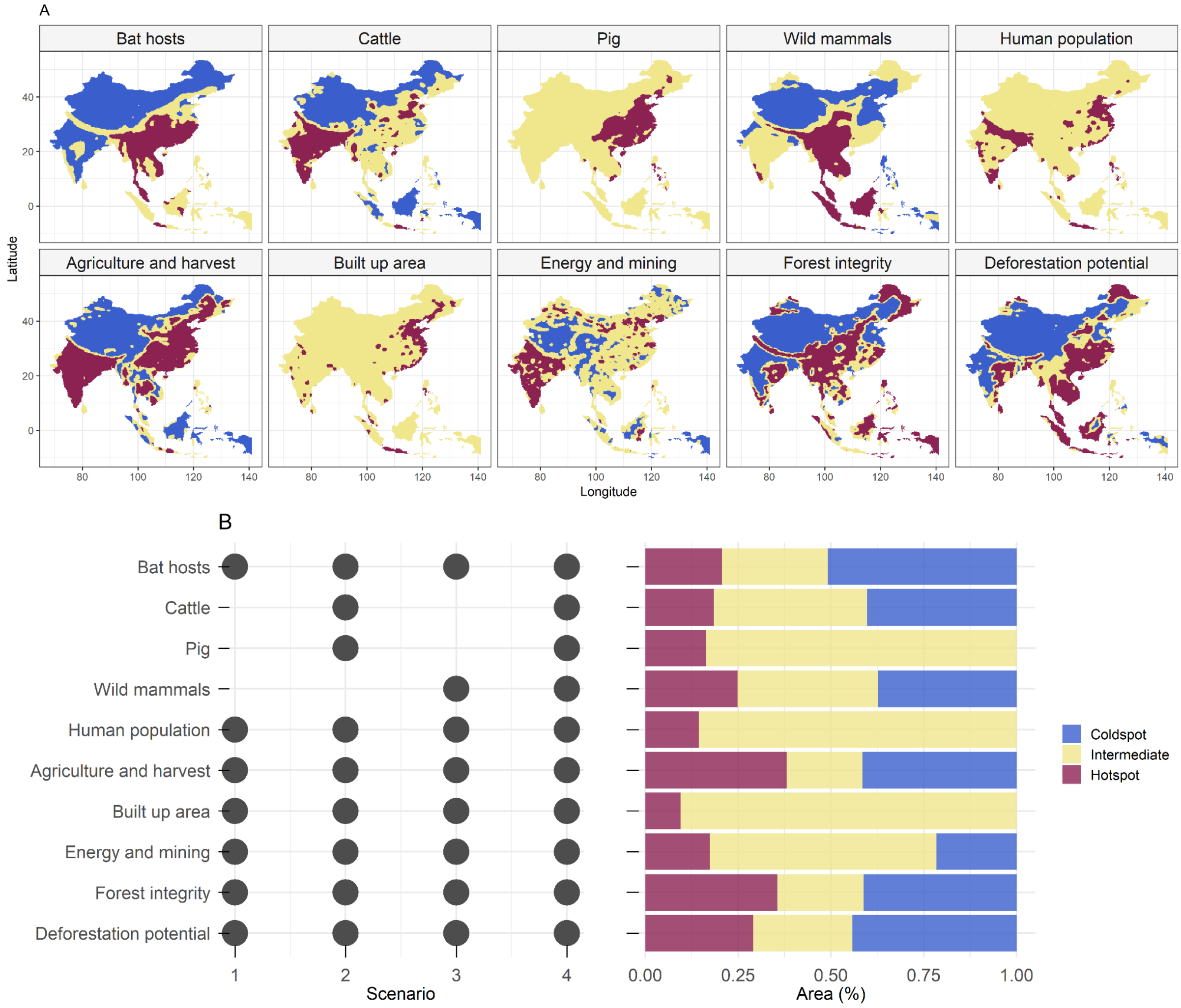


**Supplementary Fig. 1: Hotspots of potential factors contributing to emergence of SARS-like coronaviruses. A.** Spatial distribution of hotspots based on putative drivers of risk of new *Sarbecovirus* emergence evaluated in four scenarios. B. List of variables per scenario marked as black dots and proportion (%) of areas classified as hotspots, intermediate or coldspots across the study region, including wildlife, landscape change, livestock and exposure in humans. This classification used the critical value at the 0.99 percentile to define hotspots and coldspots.

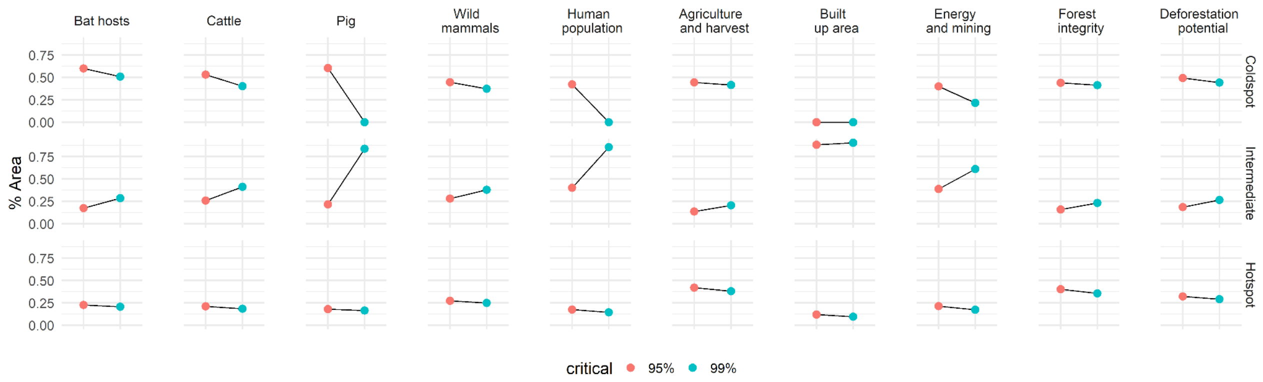


**Supplementary Fig. 2: Sensitivity analysis of hotspots of potential factors contributing to emergence of SARS-like coronaviruses at 99% and 95% critical values of quantiles for determining hotspots.** Hotspots were insensitive to change in critical values while coldspots tended to decrease and intermediate areas tended to increase.

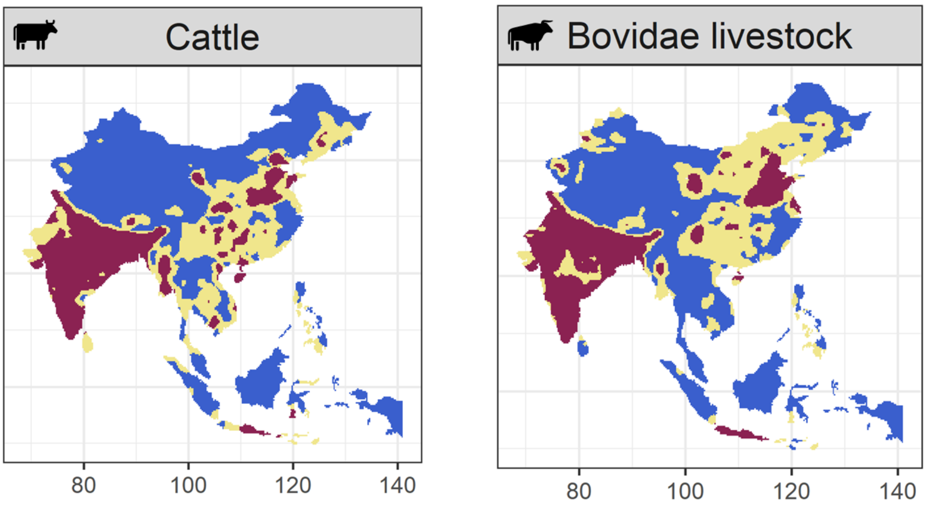


**Supplementary Fig. 3: Hotspot values for cattle and all Bovidae livestock.** Hotspots in dark red, intermediate zones in yellow, coldspots in blue.

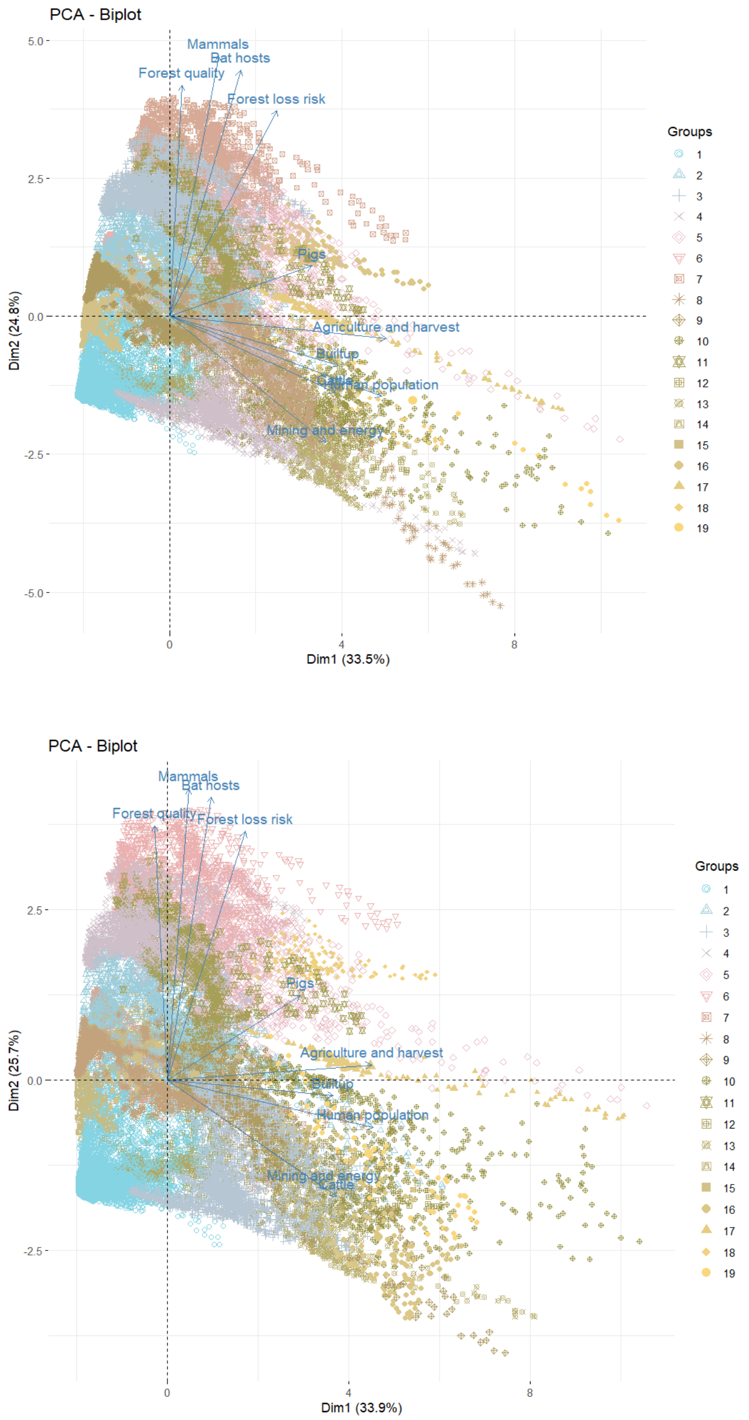


**Supplementary Fig. 4: Principal component analysis (PCA) biplot indicates variation between 19 clusters defined by multivariate spatial cluster analyses considering all variables (Scenario 4).** Upper panel: cattle-only version. Bottom panel: all Bovidae livestock version.

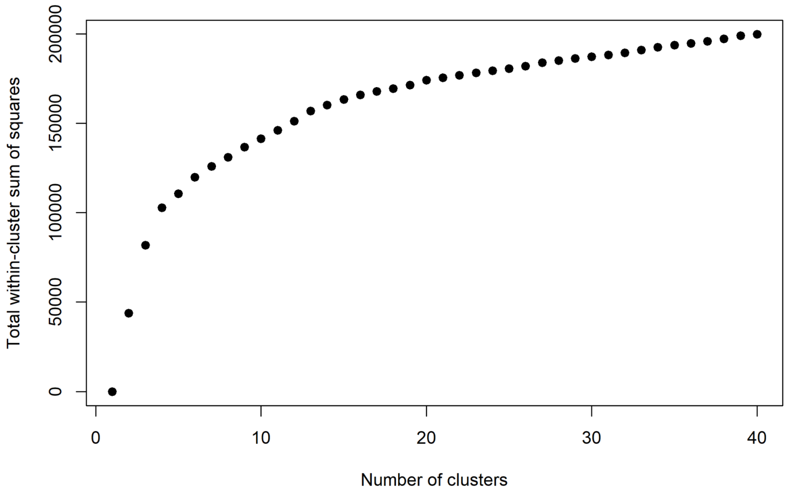


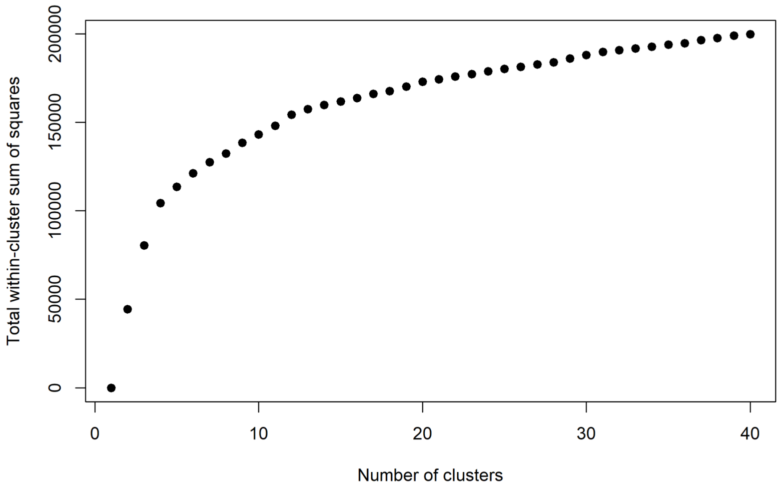


**Supplementary Fig. 5: Skater within-cluster sum of squares variation from 1 to 40 clusters for all selected variables (Scenario 4).** The optimal number of clusters informed by the max-p algorithm was 9 and 19 (respectively, for 10% and 5% human population used as minimum bound variables). Upper panel: Cattle-only version. Bottom panel: all Bovidae livestock version.

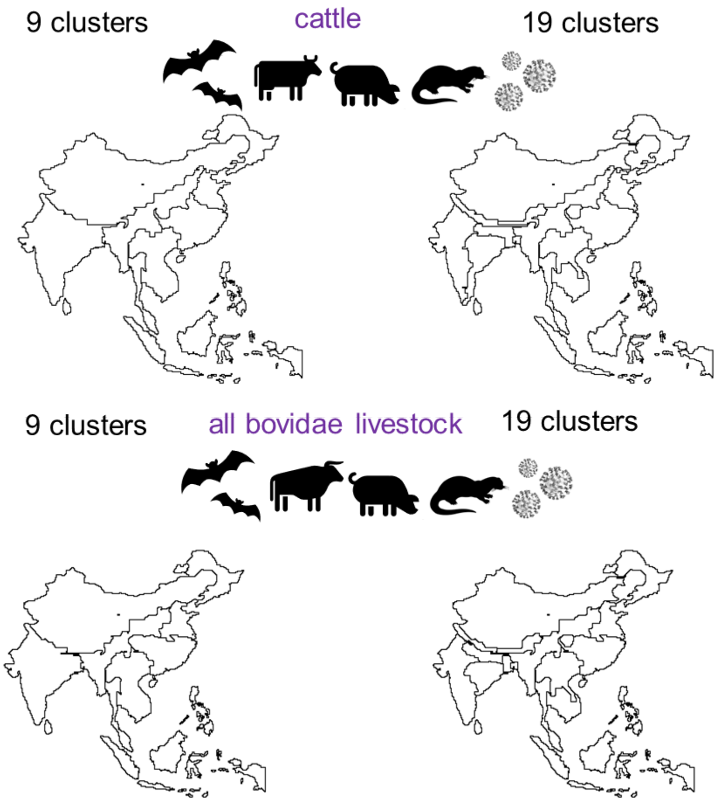


**Supplementary Fig. 6: Hierarchical nature of the spatial clusters with 9 and 19 optimal number of clusters considering the global scenario (Scenario 4).** Results presented with 19 clusters are in the main text. Upper panel: Cattle-Only. Bottom: all Bovidae livestock.

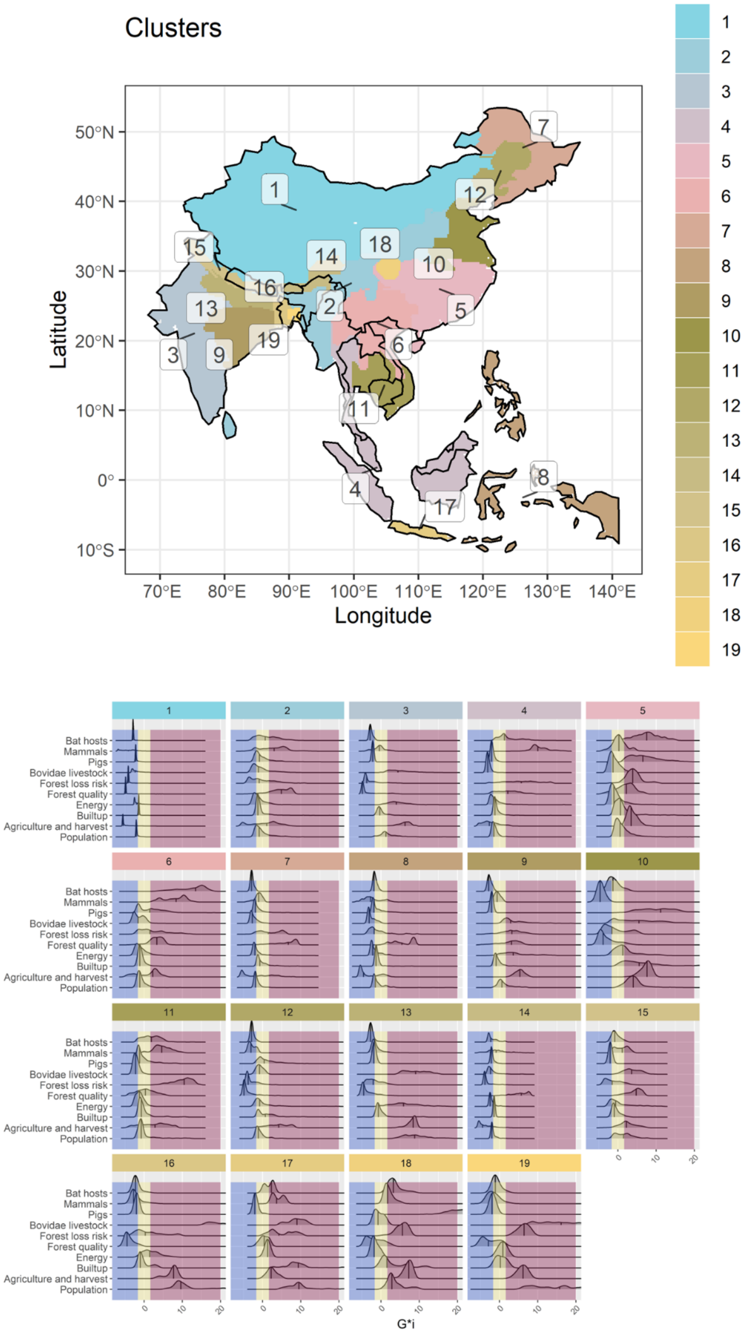


**Supplementary Fig. 7: Optimal number of multivariate clusters of all selected components associated with potentially new emerging SARS-like coronavirus (Scenario 4).** This version uses all Bovidae livestock instead of cattle-only.

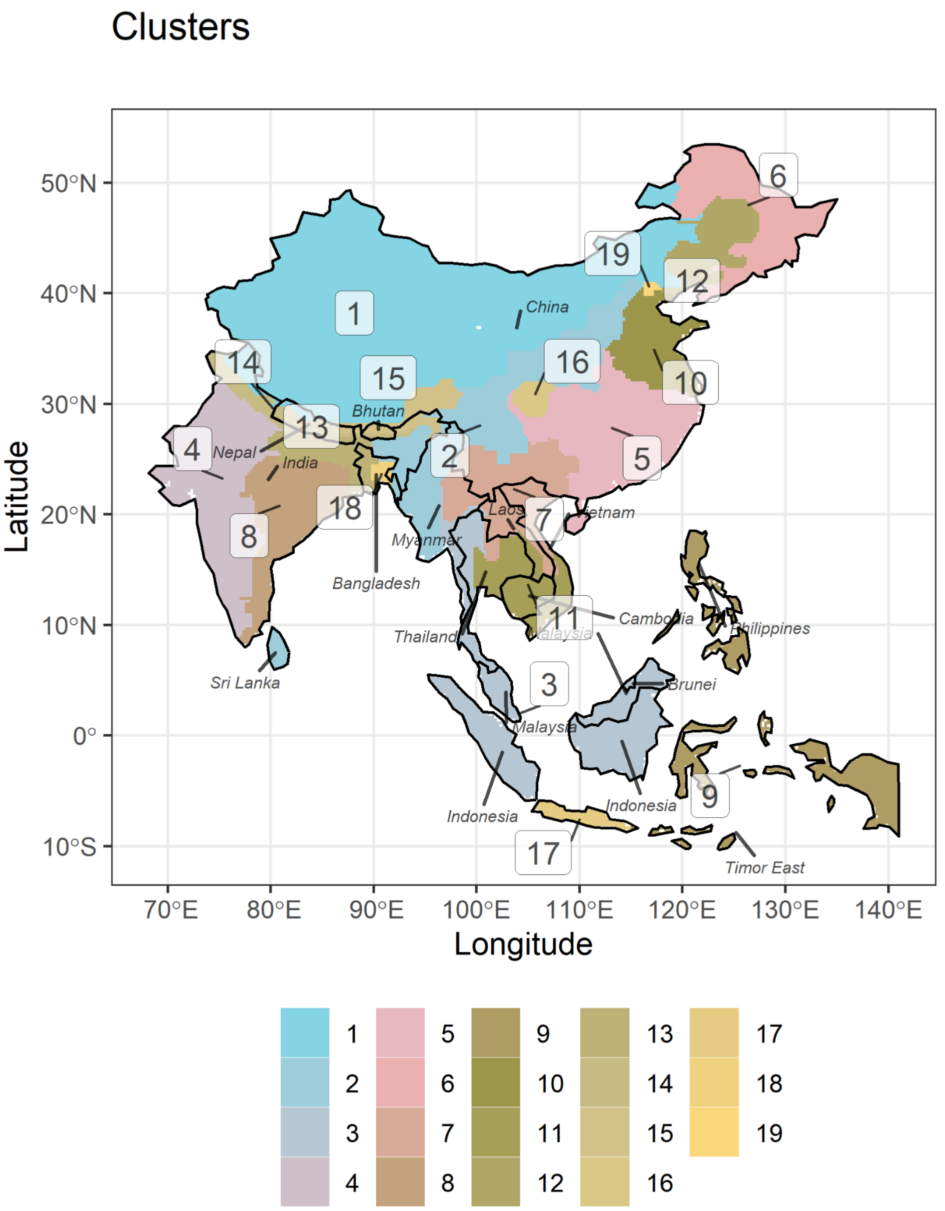


**Supplementary Fig. 8: Distribution of clusters of risk factors associated with potentially new emerging SARS-like coronaviruses. The values include all potential mammalian hosts, land use change and human exposure density distributions (Scenario 4)**. Areas located in the red zone represent hotspots, yellow zones are intermediate areas and coldspots in blue, at a 95% alpha error level.

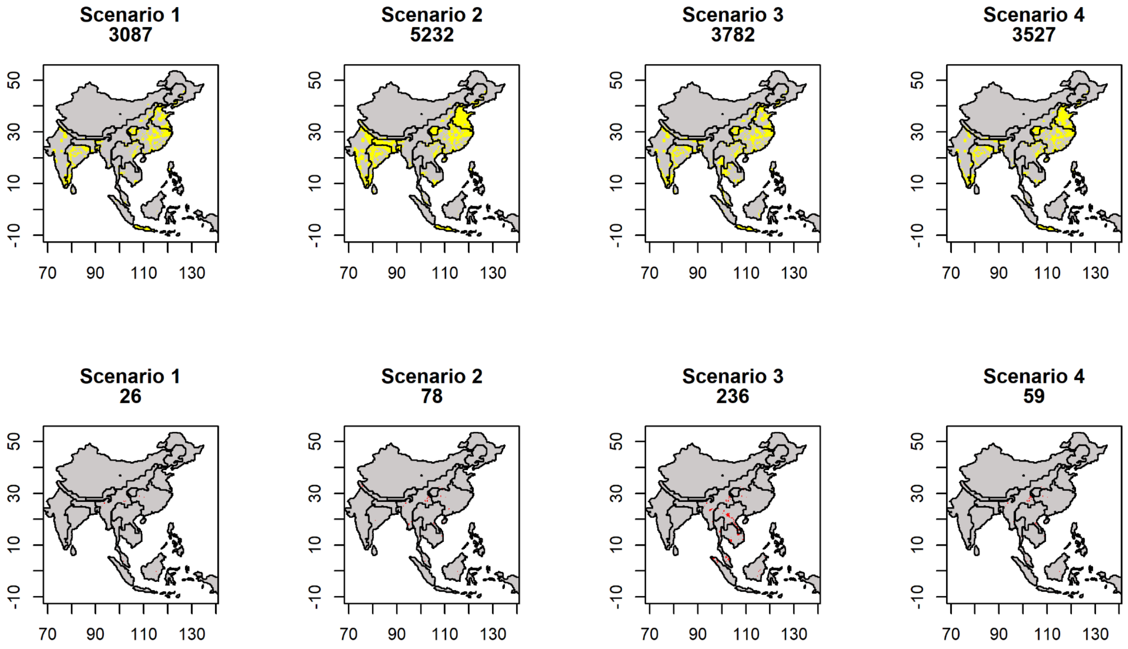


**Supplementary Fig. 9: Risk associated with transmission scenarios according to time to reach healthcare (lower and higher quantiles for healthcare access).** Boundaries in black represent the 19 clusters. Upper panel shows areas that are close from healthcare, with high hotspot overlap, in yellow. Bottom panel shows areas that are far from healthcare, with high hotspot overlap, in red. The number below every title corresponds to the grid count for the colour value. Landscape, human population and known bat hosts are included in all models, and are the sole indicators in Scenario 1, representing direct transmission. To incorporate indirect transmission through secondary hosts, mammalian livestock are included in Scenario 2, wild mammals in Scenario 3, and both mammalian livestock and wild mammals in Scenario 4.

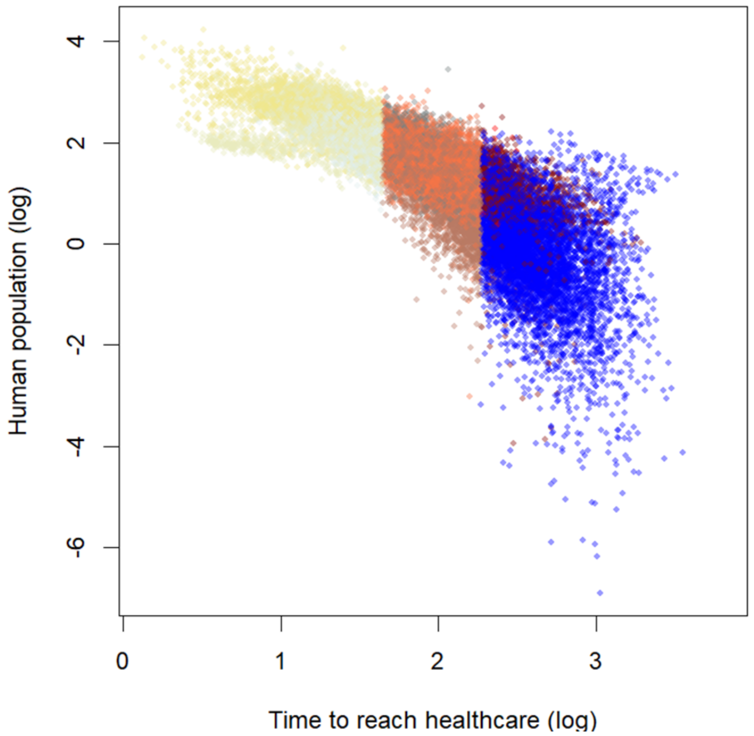


**Supplementary Fig. 10: Human population variation according to motorized travel time.** Colours represent quantiles from the bivariate map of inferred risk from Scenario 4 as a function of time to reach healthcare.

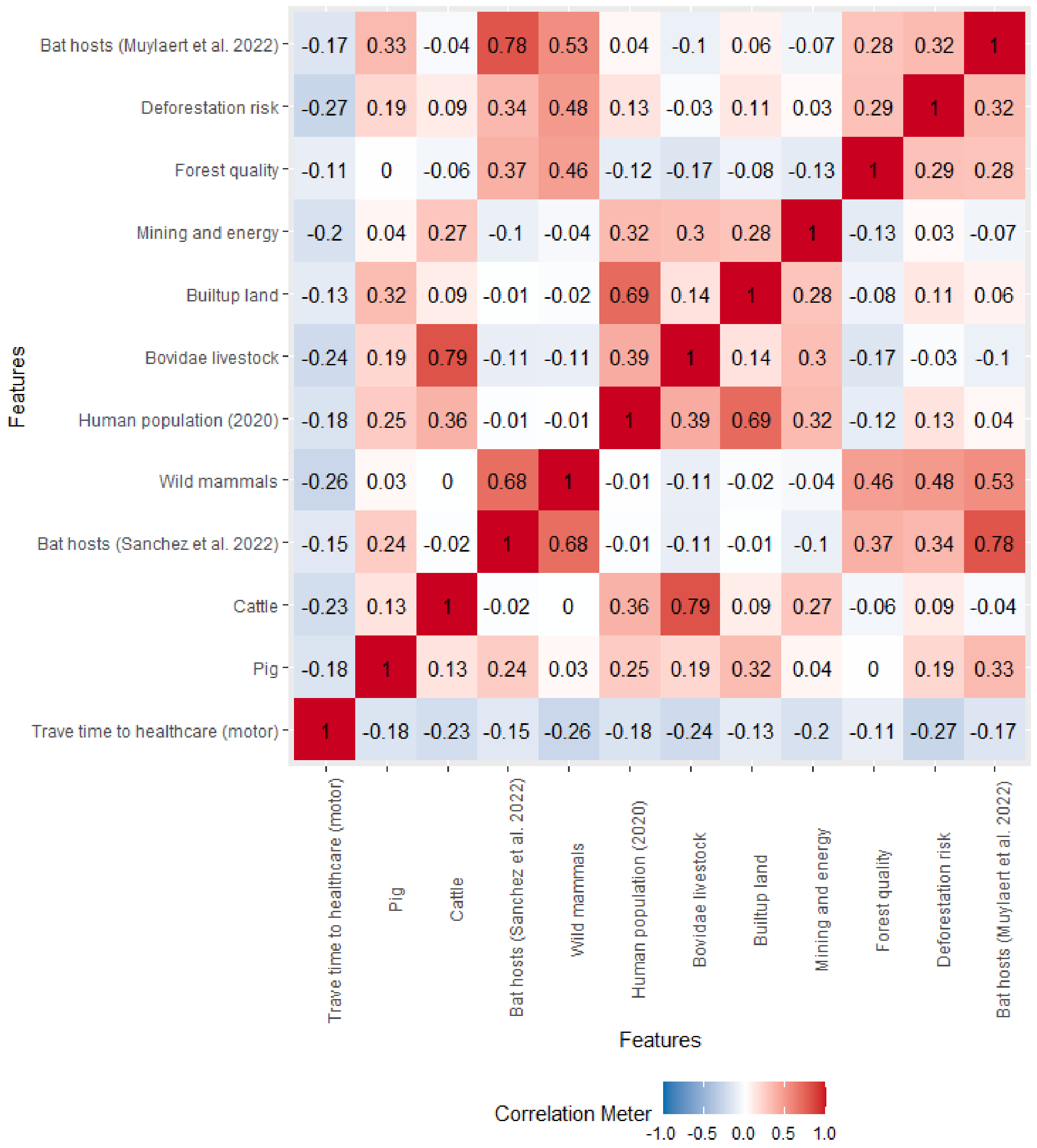


**Supplementary Fig. 11: Product-moment correlation values (*r*) of selected variables.** Known bat hosts were combined in a single layer after averaging their values. Results with the cattle-only version are displayed in the main text, and all Bovidae livestock in the supplements.

**Supplementary references**


1. Anthony, S. J. *et al.* Global patterns in coronavirus diversity. *Virus Evol* **3**, vex012 (2017).
2. Theobald, D. M. *et al.* Earth transformed: detailed mapping of global human modification from 1990 to 2017. *Earth System Science Data* **12**, 1953–1972 (2020).
3. Allen, T. *et al.* Global hotspots and correlates of emerging zoonotic diseases. *Nat. Commun.* **8**, 1–10 (2017).
4. Grantham, H. S. *et al.* Anthropogenic modification of forests means only 40% of remaining forests have high ecosystem integrity. *Nat. Commun.* **11**, 5978 (2020).
5. Shah, H. A., Huxley, P., Elmes, J. & Murray, K. A. Agricultural land-uses consistently exacerbate infectious disease risks in Southeast Asia. *Nat. Commun.* **10**, 1–13 (2019).
6. Plowright, R. K. *et al.* Land use-induced spillover: a call to action to safeguard environmental, animal, and human health. *Lancet Planet Health* **5**, e237–e245 (2021).
7. Rulli, M. C., D'Odorico, P., Galli, N. & Hayman, D. T. S. Land-use change and the livestock revolution increase the risk of zoonotic coronavirus transmission from rhinolophid bats. *Nature Food* **2**, 409–416 (2021).
8. Hewson, J., Crema, S. C., González-Roglich, M., Tabor, K. & Harvey, C. A. New 1 km Resolution Datasets of Global and Regional Risks of Tree Cover Loss. *Land* **8**, 14 (2019).
9. Sikkema, R. S. *et al.* Experimental and field investigations of exposure, replication and transmission of SARS-CoV-2 in pigs in the Netherlands. *Emerg. Microbes Infect.* **11**, 91–94 (2022).
10. Li, H. *et al.* Human-animal interactions and bat coronavirus spillover potential among rural residents in Southern China. *Biosaf Health* **1**, 84–90 (2019).
11. Zhou, P. *et al.* Fatal swine acute diarrhoea syndrome caused by an HKU2-related coronavirus of bat origin. *Nature* **556**, 255–258 (2018).
12. Gilbert, M. *et al.* Global distribution data for cattle, buffaloes, horses, sheep, goats, pigs, chickens and ducks in 2010. *Sci Data* **5**, 180227 (2018).
13. Wernike, K. *et al.* Antibodies against SARS-CoV-2 Suggestive of Single Events of Spillover to

Cattle, Germany. *Emerg. Infect. Dis.* **28**, 1916–1918 (2022).

14. Ulrich, L., Wernike, K., Hoffmann, D., Mettenleiter, T. C. & Beer, M. Experimental Infection of Cattle with SARS-CoV-2. *Emerg. Infect. Dis.* **26**, 2979–2981 (2020).
15. Nerpel, A. *et al.* SARS-ANI: a global open access dataset of reported SARS-CoV-2 events in animals. *Scientific Data* **9**, 1–13 (2022).
16. Muylaert, R. L. *et al.* Present and future distribution of bat hosts of sarbecoviruses: implications for conservation and public health. *Proc. Biol. Sci.* **289**, 20220397 (2022).
17. Sánchez, C. A. *et al.* A strategy to assess spillover risk of bat SARS-related coronaviruses in Southeast Asia. *Nat. Commun.* **13**, 4380 (2022).
18. Forero, N. *et al.* The coevolutionary mosaic of bat betacoronavirus emergence risk. *EcoEvoRxiv* (2022) doi:10.32942/osf.io/8mgv6.
19. Temmam, S. *et al.* Bat coronaviruses related to SARS-CoV-2 and infectious for human cells. *Nature* **604**, 330–336 (2022).
20. Zhang, X. *et al.* The effect of population size for pathogen transmission on prediction of COVID-19 spread. *Sci. Rep.* **11**, 18024 (2021).
21. John, R. S., Miller, J. C., Muylaert, R. L. & Hayman, D. T. S. High connectivity and human movement limits the impact of travel time on infectious disease transmission. *medRxiv* 2023.07.26.23293210 (2023) doi:10.1101/2023.07.26.23293210.
22. Winck, G. R. *et al.* Socioecological vulnerability and the risk of zoonotic disease emergence in Brazil. *Sci Adv* **8**, eabo5774 (2022).
23. Weiss, D. J. *et al.* Global maps of travel time to healthcare facilities. *Nat. Med.* **26**, 1835–1838 (2020).
