# Supplementary material for: Using drivers and transmission pathways to identify SARS-like coronavirus spillover risk hotspots
Source: Nat Commun. 2023 Oct 27;14:6854. doi: 10.1038/s41467-023-42627-2 (PMC10611769; doi:10.1038/s41467-023-42627-2)
Supplement: Supplementary file 3 — Description of Additional Supplementary Files [file 41467_2023_42627_MOESM3_ESM.pdf]

Description of Additional Supplementary Files

File Name: Supplementary Movie 1
Description: **Comparison among different scenarios of risk and access to health care.** The two data layers were divided in three quantiles and 9 unique legend colour codes.
