# Supplementary figures and images for: Using drivers and transmission pathways to identify SARS-like coronavirus spillover risk hotspots
Source: Nat Commun. 2023 Oct 27;14:6854. doi: 10.1038/s41467-023-42627-2 (PMC10611769; doi:10.1038/s41467-023-42627-2)

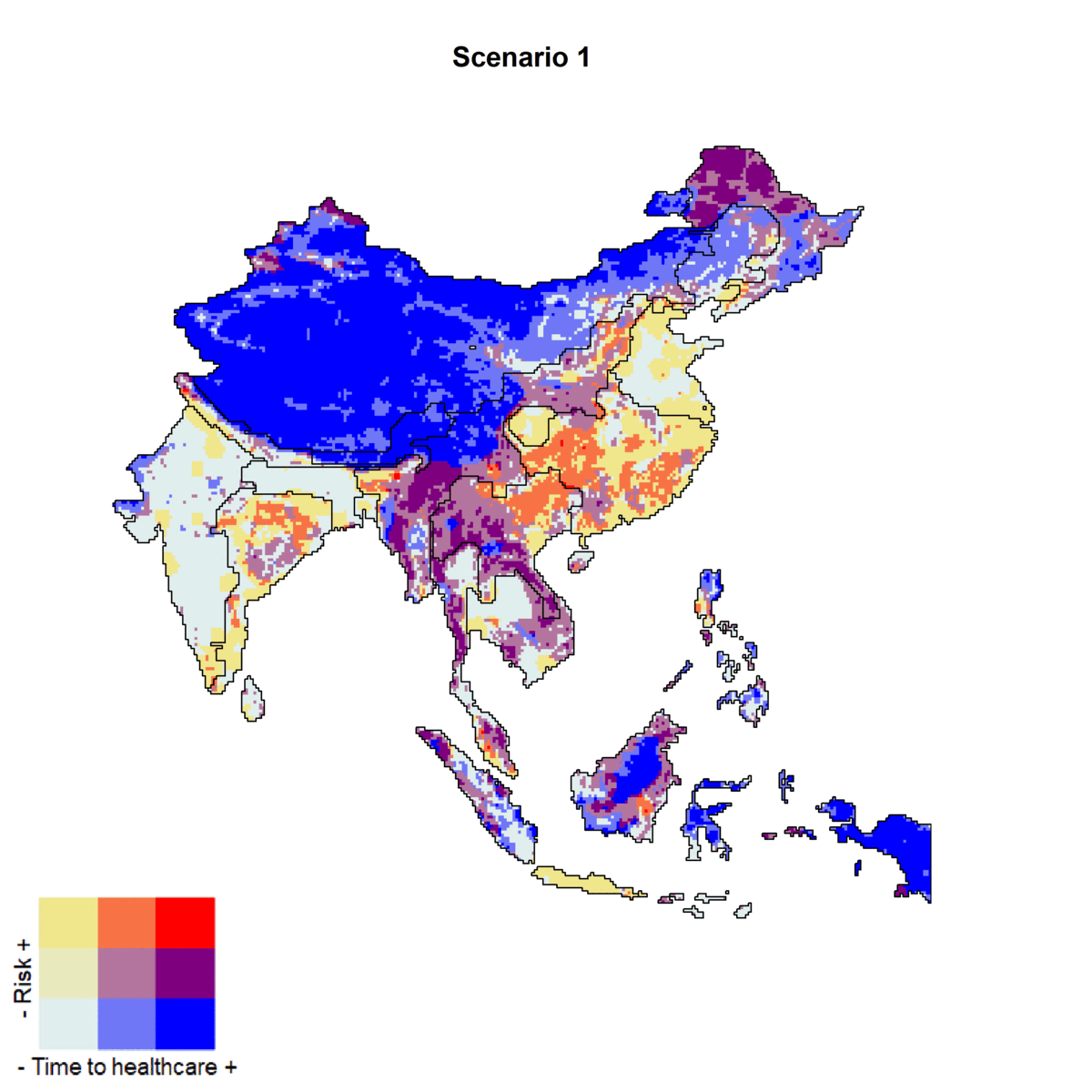

Supplement: Supplementary file 4 — Supplementary Movie 1 [file 41467_2023_42627_MOESM4_ESM.gif]
